# Repeated Cocaine Exposure Facilitates the Expression of Incentive Motivation and Induces Habitual Control in Rats

**DOI:** 10.1371/journal.pone.0061355

**Published:** 2013-04-30

**Authors:** Kimberly H. LeBlanc, Nigel T. Maidment, Sean B. Ostlund

**Affiliations:** 1 Department of Psychiatry and Biobehavioral Sciences, Semel Institute for Neuroscience and Human Behavior, University of California Los Angeles, Los Angeles, California, United States of America; 2 Brain Research Institute, University of California Los Angeles, Los Angeles, California, United States of America; University of Chicago, United States of America

## Abstract

There is growing evidence that mere exposure to drugs can induce long-term alterations in the neural systems that mediate reward processing, motivation, and behavioral control, potentially causing the pathological pursuit of drugs that characterizes the addicted state. The incentive sensitization theory proposes that drug exposure potentiates the influence of reward-paired cues on behavior. It has also been suggested that drug exposure biases action selection towards the automatic execution of habits and away from more deliberate goal-directed control. The current study investigated whether rats given repeated exposure to peripherally administered cocaine would show alterations in incentive motivation (assayed using the Pavlovian-to-instrumental transfer (PIT) paradigm) or habit formation (assayed using sensitivity to reward devaluation). After instrumental and Pavlovian training for food pellet rewards, rats were given 6 daily injections of cocaine (15 mg/kg, IP) or saline, followed by a 10-d period of rest. Consistent with the incentive sensitization theory, cocaine-treated rats showed stronger cue-evoked lever pressing than saline-treated rats during the PIT test. The same rats were then trained on a new instrumental action with a new food pellet reward before undergoing a reward devaluation testing. Although saline-treated rats exhibited sensitivity to reward devaluation, indicative of goal-directed performance, cocaine-treated rats were insensitive to this treatment, suggesting a reliance on habitual processes. These findings, when taken together, indicate that repeated exposure to cocaine can cause broad alterations in behavioral control, spanning both motivational and action selection processes, and could therefore help explain aberrations of decision-making that underlie drug addiction.

## Introduction

Many theories have been proposed concerning the mechanism by which recreational drug use transitions to addiction, a state characterized by the compulsive pursuit of drugs despite the severe negative consequences of this behavior. The incentive sensitization theory argues that extended drug use sensitizes the neural circuitry involved in assigning incentive salience to drug-paired cues, allowing these cues to exert greater control over drug-seeking behavior [1], [2]. Virtually all addictive drugs stimulate, and typically sensitize, the dopamine system [3], [4], a central mediator of incentive motivation [5]–[11]. It is proposed that drug-induced adaptations in the dopamine system render it hypersensitive to drugs and their associated cues, allowing these stimuli to elicit intense drug craving and trigger drug seeking.

It has also been proposed that chronic drug use causes a transition in the systems controlling drug-seeking behavior [12]–[16]. This account is based on research showing that rodents performing an instrumental action (e.g., pressing a lever) for food reward rely on two competing strategies: a goal-directed (or action-outcome) strategy that involves considering the consequences of potential actions (i.e., whether that consequence is desirable or to be avoided), and a habitual (or stimulus-response) strategy that involves reacting – without deliberation – to prevailing stimuli that have acquired the ability to trigger certain actions [17]. Importantly, the control of actions shifts over the course of training, with goal-directed control dominating early in training and habitual control taking over as the action becomes firmly established. Interestingly, recent animal studies have shown that drug-seeking actions undergo a more rapid transition to habitual control than food-seeking actions [18], [19], and generally display characteristics of habitual control, like insensitivity to changes in goal value [20], [21].

Not surprisingly, many studies investigating how cues alter drug-seeking behavior have used drug self-administration tasks [21], [22], in which a drug serves as the reinforcing stimulus, since this most closely models drug seeking in humans. Interestingly, it has been shown that drug self-administration levels are elevated in animals previously given repeated noncontingent drug exposure [23], [24], even when the pre-exposed drug is delivered intracranially [25], [26]. While such studies suggest that drug pre-exposure can alter drug seeking, in such situations it is difficult to pinpoint the source of this effect because the drug may be having multiple effects on behavior. For instance, the drug is likely to have direct, action-specific effects because it serves as the reinforcer or goal of behavior. But it may also have broader, nonspecific effects on behavior, perhaps by sensitizing the incentive motivational system or by biasing action selection to favor habitual performance. In order to selectively target a drug's ability to produce broader changes in behavior it is useful to use food, rather than the drug itself, as the reinforcing stimulus (see [27]). Importantly, there is considerable evidence that repeated exposure to drugs can have long-lasting effects on the control of actions motivated by natural rewards like food [28]–[38]. For instance, previous studies have shown that rats given repeated exposure to the psychostimulant amphetamine show a heightened sensitivity to the incentive motivational effects of food-paired cues [28] and exhibit accelerated habit learning [29], [30]. However, the impact of cocaine, another widely abused psychostimulant, on these phenomena has not been as well characterized. While both cocaine and amphetamine are known to cause persistent changes in behavior and neurotransmission, they have distinct modes of action on dopamine signaling and appear to differentially engage and induce adaptations in components of circuitry underlying learning and motivation [4], [39]. In fact, with doses that result in similar behavioral psychoactive effects, amphetamine produces greater dopamine release in the caudate and accumbens [40], and more diffuse release within the accumbens than cocaine [41]. If such differences translate into differences in the induction of sensitization in the dopamine system, one might expect the behaviors that this system supports to be differentially affected by amphetamine and cocaine. Specifically, it is possible that cocaine's more modest effects on the dopamine system would lead to less substantial alterations in incentive motivation and/or habit formation, when compared to alterations generated by amphetamine. In this study, we investigated whether rats repeatedly exposed to cocaine exhibit either enhanced incentive motivation for food reward or a bias towards habitual control.

## Methods

A timeline for Experiment 1 is provided in Table 1.

**Table 1:** Experimental timeline: Experiment 1.

Phase	Procedure	Days
Instrumental training	Action 1→Reward 1	1–14
Pavlovian training	CS+→Reward 1CS−→No Reward	15–28
Drug treatment	Cocaine (15 mg/kg)or Saline	29–34
Withdrawal	Remain in home cage	35–44
Retraining	Action 1→Reward 1	45–48
Pavlovian-to-instrumental transfer test	Deliveries of CS+ and CS− with access to Action 1	49
Instrumental conditioning	Action 2→Reward 2	50–53; 55
Devaluation testing	Reward 2 devalued or not prior to test with Action 2	54, 56

### Ethics statement

All procedures were approved by the UCLA Institutional Animal Care and Use Committee, and were performed in accordance with the National Research Council's *Guide for the Care and Use of Laboratory Animals*.

### Subjects and apparatus

Male Long Evans rats (mean weight: 330±10.13 g) were used as subjects. Rats were group housed in a climate-controlled vivarium and were tested during the light phase of the light/dark cycle (lights on from 7am to 7pm). Rats had **ad libitum** access to tap water throughout the study and were food deprived (10–14 g of chow per day) to maintain them at ∼85% their free-feeding body weight. Rats were trained in 8 identical Med Associates (East Fairfield, VT) operant chambers housed within sound- and light-resistant shells. The chambers contained two retractable levers that could be inserted to the left and right side of a recessed food cup on one end wall. A 3-W, 24-V houselight mounted on the top center of the opposite end wall provided illumination. The chambers were also equipped with a tone generator and a clicker.

### Drugs

Cocaine hydrochloride, provided by the National Institute on Drug Abuse Drug Supply Program, was dissolved in sterile saline (0.9% NaCl) and filter-sterilized prior to injection.

### Instrumental Training

Rats were first given two magazine training sessions in which they received 20 grain-based food pellets (45 mg, Bioserv, Frenchtown, NJ) on a fixed time 1-min schedule. This was followed by 14 d of instrumental training, consisting of 30-min sessions with constant access to an active and inactive lever. Pressing on the active lever (left or right; counterbalanced with Pavlovian training and cocaine exposure conditions) resulted in the delivery of grain pellets, while pressing on the inactive lever was without consequence. The schedule of reinforcement used for the active lever progressed through consecutive days of continuous reinforcement, random interval (RI) 5s, RI-15s, RI-30s, followed by 10 d of RI-45s. Two subjects failed to discriminate between the two levers (<90% total presses on the active lever during the last day) and were excluded from the rest of the study.

### Pavlovian Training

Rats were then given 14 daily 30-min sessions of Pavlovian training. During the first 11 sessions, the presentation of one of two auditory stimuli (CS+; either a 3 kHz, 75 dB tone or a 2 Hz, 75 dB click, 30-s duration) was followed by delivery of 3 grain pellets at the offset of the cue; 10 CS+ presentations were delivered on a variable time 2-min schedule. The last 3 sessions were the same as the first 11 sessions, but with the addition of two non-reinforced presentations of the alternative auditory stimulus (CS−) during the middle and end of the sessions. Limited CS− exposure is commonly used in PIT studies to habituate subjects to the unpaired cue while minimizing their experience with the explicitly unpaired cue-reward contingency, which can itself support unintended learning (for discussion, see [42]). Magazine entries were recorded to monitor acquisition of conditioned approach behavior.

### Cocaine sensitization

In experiment 1, rats were divided into two groups: a cocaine exposure group (n = 11) and a saline exposure group (n = 13) receiving 6 once-daily intraperitoneal (IP) injections of 15 mg/kg cocaine HCl or saline (1 ml/kg), respectively, before being placed in the behavioral chambers (with the houselight on) for 45 min and subsequently returned to their home cages. Both levers were retracted during these sessions, and no stimuli (rewards or cues) were delivered. Rats were then given 10 d of cocaine (or saline) withdrawal, during which they remained undisturbed in their homecages. The sensitization protocol was based on an earlier study finding sensitization of cue-evoked reward seeking in rats given repeated amphetamine injections [28]. For cocaine, similar dosage, duration, and abstinence parameters have been shown to support other forms of behavioral sensitization [43]–[45]. However, a second experiment (experiment 2) was conducted to confirm that this cocaine exposure regimen is, indeed, effective in producing a locomotor sensitization effect. For this experiment, the behavioral chambers were equipped with an activity sensor (model AS 2024,O'Hara & Co, Tokyo, Japan) to measure general activity levels during 45-min post-injection exposure sessions. As in experiment 1, rats received 6 d of exposure, with one group (n = 12) receiving 15 mg/kg cocaine injections before being placed in the chamber and a second group (n = 12) receiving saline injections. For both experiments, magazine entries were continuously monitored throughout these exposure sessions.

### Pavlovian-to-instrumental transfer (PIT) testing

Subjects were retrained for 3 d on the instrumental response on a RI-45s schedule. On the following day, rats received a 30-min extinction session in which both levers were available but produced no rewards. During the PIT test, both levers were extended into the chamber and were retracted at the end of the test. All lever presses were recorded during this session but no rewards were delivered. The two 30-s auditory cues (CS+ and CS−) were non-contingently presented 4 times each in alternation (tone, click) to assess their ability to influence lever press performance. The number of presses performed on each lever during the min before the cue onset was used as the Pre-CS, or baseline, response rate. The magazine entry detector in one of the operant chambers was not functioning properly, and the data for two vehicle subjects was lost.

### Instrumental Retraining

Following the PIT test, subjects were retrained on the previously inactive lever for a new outcome (sucrose or chocolate purified pellet, 45 mg, Bioserv) over four daily sessions, each ending after 30 min or once 30 pellets had been earned, whichever came first. This was done to establish a new instrumental action that was likely to be performed using a goal-directed strategy, so that we could investigate whether cocaine pre-exposure altered the use of such a strategy. Lever pressing was reinforced on a continuous schedule on day 1 and an RI-30s schedule thereafter. To ensure equal exposure to the other (control) pellet type, rats were allowed to consume 30 of these pellets (presented in a stainless steel cup) in an alternative context similar to their homecages either 30-min before or immediately after each instrumental training session, alternating over days.

### Devaluation test

We used a specific satiety outcome devaluation procedure to assess the rats' ability to adjust their lever pressing according to a change in outcome value. This procedure induces transient changes in reward palatability [46] and preference [47], [48], and is an effective tool for reversibly altering the incentive value of rewards to detect changes in reward-seeking [49], [50]. Subjects were given unlimited access (>30 g) to either the pellet used to reinforce lever pressing (for the devalued test) or the other control pellet that they were exposed to during the training phase but never earned through an instrumental contingency (for the nondevalued test) in an alternative context for 1 h before each test. Test sessions began with 5 min of extinction during which the lever was available but did not produce reward. This was immediately followed by a 25-min rewarded phase during which lever pressing was reinforced with the outcome previously delivered by that action on a RI-30s schedule. Thus, we assessed the sensitivity of instrumental performance to reward devaluation in the absence (extinction test) and in the presence (rewarded test) of response-contingent feedback about the current value of the training outcome. After one day of retraining on the lever using an RI-30s schedule, the second devaluation test was administered with rats fed to satiety on the other pellet type (either the pellet used during training or the control pellet, whichever was not used in Test 1). One subject in the vehicle group responded for the devalued outcome to a statistically anomalous degree (Chauvenet's criterion <0.5) and was excluded from the analysis. Another subject from the vehicle group died between test 1 and test 2 and his data was also omitted from the devaluation tests.

### Data analysis and statistics

Data from the PIT test were calculated as an elevation ratio (CS/(pre-CS+CS)), which reflects the change in responding (either presses or magazine entries) during the cue relative to the total responses performed during the baseline and cue periods. An elevation ratio of 0.5 occurs if lever pressing during the cue and pre-cue baseline periods are equal, and more lever pressing during the cue presentation results in an elevation ratio >0.5. Data from the extinction and rewarded portions of the devaluation tests were analyzed as a percentage of baseline response rates, which were taken from the final training session before each test. Data were analyzed with mixed ANOVAs using within- and between-subjects factors as appropriate. For reward devaluation testing, the analysis also included test order (which outcome was devalued first) as a covariate.

## Results

### Pre-training

**Figure 1:**
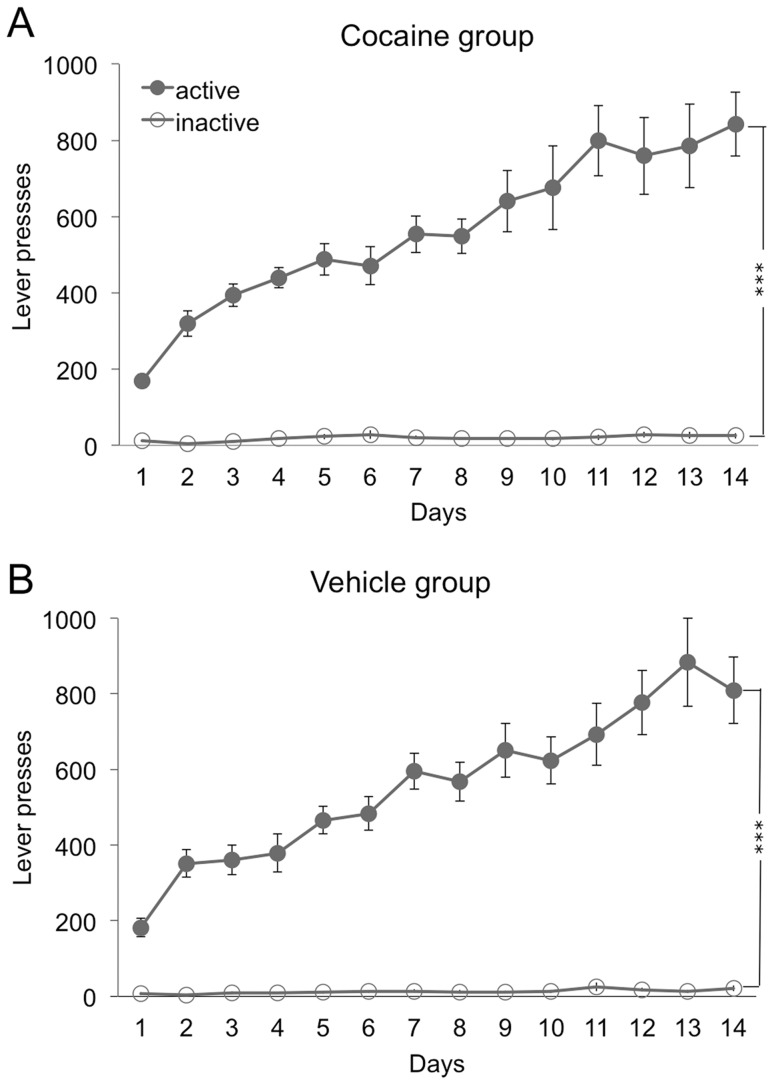
Instrumental training. Instrumental training on the active and inactive lever, shown as average lever presses over days displayed separately for the cocaine group (1A) and the vehicle group (1B). Means +/− SEM. *** = p<0.001.

**Figure 2:**
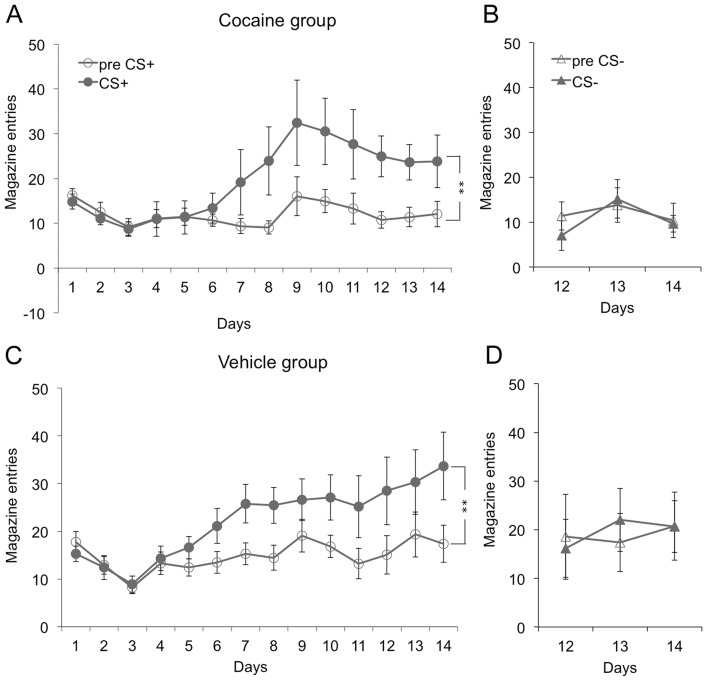
Pavlovian training. Pavlovian training, shown as magazine entries made in response to the CS and in the 30 s period immediately before it (preCS), displayed separately for CS identity and group. CS+ trials for the cocaine group (2A) and the vehicle group (2C) are shown next to CS− trials for the cocaine group (2B) and vehicle group (2D). Means +/− SEM. ** = p<0.01.

In experiment 1, rats acquired the instrumental response rapidly (see Figure 1), distinguishing between the active and inactive lever from the first training session. A lever×day×group ANOVA revealed a significant main effect of lever (*F* (1,22) = 261.26, *p*<0.001) and day (*F* (13,286) = 33.47, *p*<0.001) and a significant lever by day interaction (*F* (13, 286) = 31.11, *p*<0.001), indicating that rats further learned to distinguish the active lever from the inactive lever over days. There were no significant interactions with group (largest F value: *F* (13,286) = 0.6, *p*>0.05), and no group effect (*F* (1,22) = 0.025, *p*>0.05). Figures 2a and 2c shows that rats displayed higher levels of magazine approach behavior during the CS+ compared to the pre-CS+ period during Pavlovian training. The period×day×group ANOVA found a significant main effect of period (*F* (1,19) = 16.31, *p* = 0.001) and day (*F* (13, 247) = 5.67, *p*<0.001) and a significant period by day interaction (*F* (13,247) = 8.61, *p*<0.001). There were no interactions with group (largest F value: *F* (13,247) = 1.1, *p*>0.05), and no main effect of group (*F* (1,19) = 0.534, *p*>0.05). Both groups also learned to discriminate between the CS+ and CS−, as shown in Figure 2b and 2d. A period×CS×day×group ANOVA detected a significant main effect of CS (*F* (1,20) = 13.61, *p* = 0.001) and period (*F* (1,20) = 17.5, *p*<0.001) and a significant CS by period interaction (*F* (1,20) = 14.19, *p* = 0.001), representing greater approach behavior to the CS+ than the CS−, and greater approach during the CS+ than during the pre-CS+ period. There were no other significant interactions (*F* (2,40) = 2.16, *p*>0.05), and no main effect of group (*F* (1,20) = 1.5, *p*>0.05).

### Pavlovian incentive motivation testing

**Figure 3:**
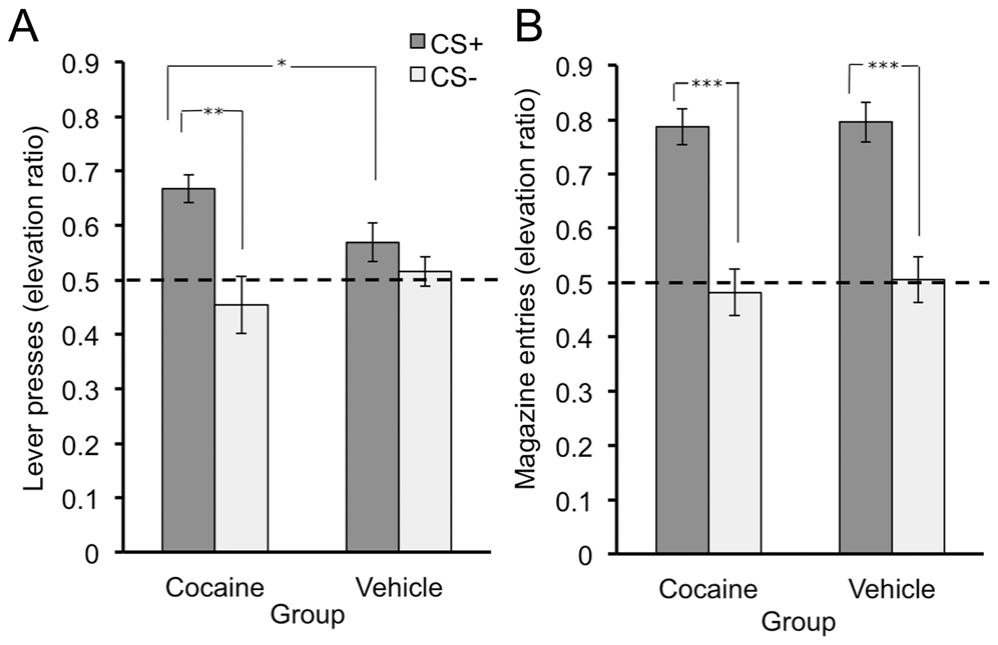
Pavlovian-to-instrumental transfer test results. PIT test results, calculated as an elevation ratio for both lever presses (3A) and magazine entries (3B) for the cocaine and vehicle groups. The dashed line at 0.5 represents the baseline level of responding. Means +/− SEM. * = p<0.05, ** = p<0.01, *** = p<0.001.

Rats in experiment 1 were then given the cocaine administration and abstinence procedures, which was followed by PIT testing to assess the influence of the reward-paired cue on their instrumental performance. The conditioning and PIT testing parameters, modeled after a similar study [51], were carefully selected to ensure that normal rats would show minimal levels of cue-motivated behavior. We reasoned that these suboptimal conditions would facilitate detection of an enhancement of the PIT effect in cocaine-treated rats. Indeed, as illustrated in Figure 3a, the cocaine-exposed group did show a significant increase in lever pressing during the CS+ but not during the CS−, while the vehicle group's lever pressing did not appear to be affected by either cue. A CS×group ANOVA revealed a significant main effect of CS (*F* (1,22) = 11.80, *p* = 0.002) as well as a CS by group interaction (*F* (1,22) = 4.23, *p* = 0.05), but no main effect of group (*F* (1,22) = 0.341, *p*>0.05). Separate analysis of the data from each group found a significant effect of CS for the cocaine-exposed group (*F* (1,10) = 10.93, *p*<0.01) but not for the vehicle group (*F* (1,12) = 1.34, *p*>0.05), and direct comparison of CS+ responding between the two groups revealed that the elevation in pressing was significantly greater for the cocaine-treated rats (unpaired t-test: *t* (22) = 2.25, *p*<0.05). Baseline (pre-CS) press rates (responses per minute) did not significantly differ (cocaine group: - 3.10±0.42, vehicle group: - 5.17±0.99; *t* (22) = −1.80, *p*>0.05). Though vehicle treated rats did not show a significant PIT effect, they did show elevated magazine approach behavior during the CS+, relative to the pre-CS period, as did cocaine treated rats (Figure 3b). A CS×group ANOVA found a significant main effect of CS (*F* (1,20) = 69.1), *p*<0.001), but no CS by group interaction (F (1, 20) = 0.041, p>0.05) or main effect of group (*F* (1,20) = 0.127, *p*>0.05). Baseline magazine entry rates (responses per minute) did not significantly differ between groups (cocaine group - 4.69±0.87, vehicle group - 4.86±1.03; *t* (20) = −0.122, *p*>0.05). Thus, both groups showed anticipatory conditioned responding to CS+, revealing that the lack of a PIT effect in the vehicle group was not due to a general impairment in Pavlovian conditioning.

### Habitual control testing

**Figure 4:**
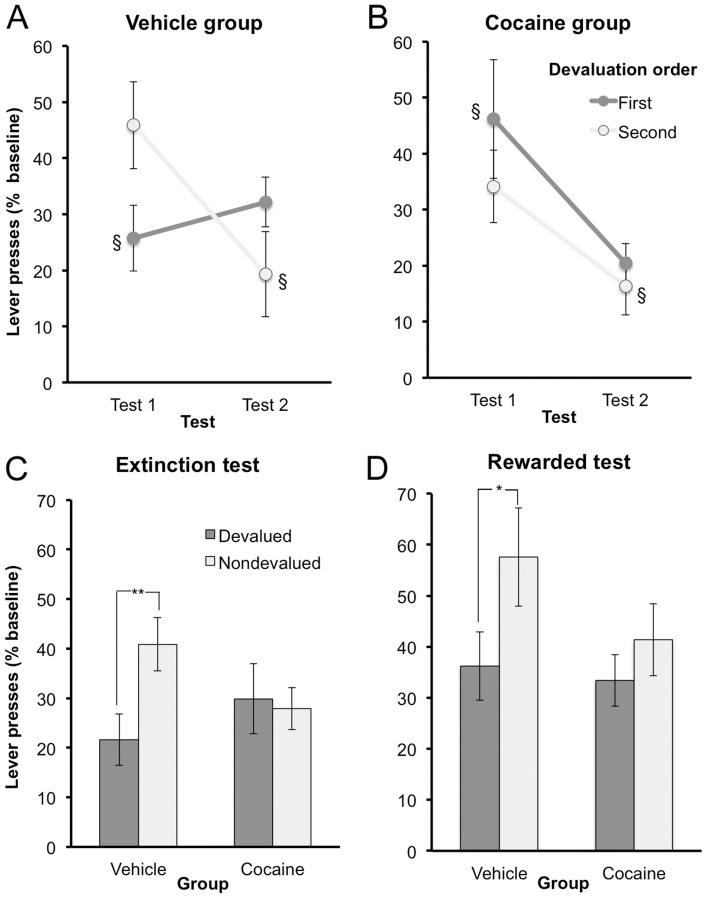
Outcome devaluation test results. Figure 4 A–D depicts lever pressing during the devaluation tests, represented as a percentage of baseline responding during training. Figure 4 A–C display results when animals are tested in extinction conditions, whereas Figure 4 D displays results when animals are tested in rewarded conditions. Figure 4A and 4B illustrate the lever presses during the test plotted separately for each group (vehicle – 4A, cocaine – 4B). ‘First’ represents the subset of animals that had the trained outcome devalued in Test 1, whereas ‘Second’ represents the subset of animals that had the trained outcome devalued in Test 2. This symbol - § - indicates the data point at which the trained outcome was devalued (i.e. the devalued test condition). Figures 4C and 4D display the results for the extinction portion of the test (4C) and rewarded portion of the test (4D) collapsed across tests and plotted separately for test condition: trained outcome devalued (devalued) vs. control outcome devalued (nondevalued). Means +/− SEM. * = p<0.05, ** = p<0.01.

Rats from experiment 1 were then retrained on the previously inactive lever for a novel outcome. There was no difference between the two groups in their acquisition of lever pressing, as similar levels of total lever presses were apparent on the last day (cocaine group (n = 11): 307.55±44.24, vehicle group (n = 11): 394±44.58). A day×group ANOVA revealed a significant main effect of day (*F* (4,80) = 73, *p*<0.001), but no day by group interaction (*F* (4,80) = 2.04, *p*>0.05) or main effect of group (*F* (1,20) = 0.002, *p*>0.05). As previously mentioned, rats were familiarized with the “control” pellet by giving them an equivalent quantity of control pellets to consume either preceding or following the training sessions. An ANOVA conducted on the last two days of training using exposure time (before or after session, within subjects) and group as factors found no main effects or interaction between these variables (all F values <1). To assess habit formation, we then conducted outcome devaluation tests to determine the degree to which their performance of this new response was dependent on the current value of the training outcome. All rats were tested once after devaluing the pellet used during training (devalued test) and a second time after devaluing the control pellet (nondevalued test), with test order balanced across groups. The first phase of outcome devaluation testing was conducted in extinction (i.e., without response-contingent feedback about the current value of the training outcome). Although this procedure is critical for probing the dependence of lever press performance on the subjects' memory of the action-outcome contingency and outcome value, an unfortunate consequence of this procedure is that repeated extinction testing produces its own suppressive effects on task performance. We therefore included test (test 1 and test 2) and devaluation order (whether the pellet used to reinforce lever pressing was devalued in test 1 or test 2), as well as group (cocaine vs. vehicle), in a mixed ANOVA to evaluate the contributions of each of these factors to test performance. This analysis found a significant main effect of test (F (1,18) = 13.02, p<0.01), with less responding in test 2 than test 1, presumably resulting from extinction generated during test 1. The ANOVA also revealed a three-way interaction between test, devaluation order, and group (F (1,18) = 5.40, p<0.05), but found no other significant main effects or interactions (largest F value: F (1,18) = 2.03, p>0.05). Above and beyond the influence of test order (i.e., extinction) on performance, this three-way interaction suggests that the groups differed in their sensitivity to outcome devaluation; specifically, whereas the vehicle group's rate of responding in any given test depended on whether or not their training outcome was currently devalued, this did not appear to be true for the cocaine group. To evaluate this account, we conducted separate test by devaluation order ANOVAs on the data from each group. A significant main effect of test was found for both the vehicle group (*F* (1,9) = 5.33, *p*<0.05) and the cocaine group (F (1,9) = 8.26, p<0.05), confirming that extinction was affecting performance in both conditions. More importantly, the vehicle group also exhibited a significant test by devaluation order interaction (F (1,9) = 14.36, p<0.01), which was not significant for the cocaine group (F (1,9) = 0.27, p>0.05) (see Figures 4a and 4b, respectively). No other effect or interaction reached significance. Figure 4c shows these data plotted according to whether the training outcome was devalued or not at test, ignoring test order and, consequently, the effects of extinction. A devaluation by group ANOVA (using test order as a covariate) found no effect of devaluation (F (1,18) = 1.23; p>0.05) or group (F (1,18) = 0.002; p>0.05), but did detect a devaluation by group interaction (F (1,18) = 4.34; p = 0.05). As can be seen, the vehicle group exhibited goal-directed control, decreasing their lever pressing during the devalued test, relative to the nondevalued test (t (10) = −3.84, p = 0.003). The cocaine group, on the other hand, showed insensitivity to devaluation, responding at similar levels in devalued and nondevalued tests, a profile indicative of habitual performance (t (10) = 0.201, p>0.05).

The extinction test phase was immediately followed by a rewarded phase, during which rats were given response-contingent feedback about the current value of the reward. Our initial inspection of the data from this test indicated that, for most rats, sensitivity to devaluation (i.e., a suppression in responding in the devalued test relative to the nondevalued test) was most apparent in the first 10 min of the rewarded phase, presumably because rats were becoming sated on the training outcome during the nondevalued test session. Therefore, we focused our analysis on the first 10 min of this 25-min test (Figure 4d). A devaluation×group ANOVA found a significant main effect of devaluation (*F* (1,20) = 4.60, *p*<0.05) but found neither a devaluation by group interaction (*F* (1,20) = 0.96, *p*>0.05) nor a main effect of group (*F* (1,20) = 1.51, *p*>0.05). While this lack of an interaction or main effect of group indicates that the two groups did not significantly differ in their sensitivity to response-contingent feedback, inspection of the data in Figure 4d suggests that this sensitivity was at least numerically more apparent in the performance of saline-treated rats. Furthermore, ANOVAs conducted separately for each group detected a significant effect of devaluation in the vehicle group (*F* (1,10) = 6.53, *p*<0.05), but no effect in the cocaine group (*F* (1,10) = 0.54, *p*>0.05).

### Behavioral sensitization

**Figure 5:**
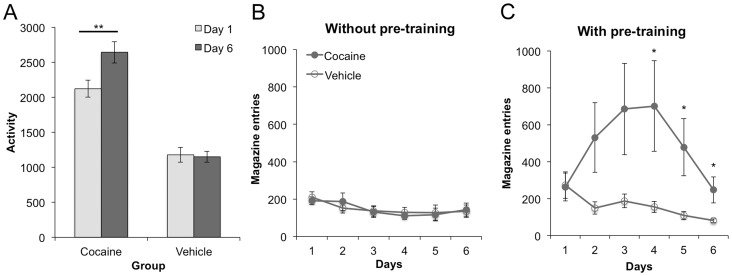
Cocaine sensitization in experiments 1 and 2. 5A – Locomotor activity on days 1 and 6 for the cocaine and vehicle groups from experiment 2. 5B – Magazine entries during cocaine sensitization for the cocaine and vehicle groups from experiment 2, in which there was no pre-training before sensitization. 5C – Magazine entries during cocaine sensitization for the cocaine and vehicle groups from experiment 1, in which there was pre-training before sensitization. Means +/− SEM. * = p<0.05, ** = p<0.01.

In experiment 2, we found that the cocaine exposure regimen used in experiment 1 was effective in producing locomotor sensitization, such that cocaine-treated rats showed an increase in activity over treatment days, whereas vehicle-treated rats did not (see Figure 5a). A mixed ANOVA with day (1 vs. 6) as the within subjects factor and group (cocaine vs. vehicle) as the between subjects factor found a significant main effect of day (F (1,22) = 8.28, p<0.01), a significant main effect of group (F (1,22) = 73.01, p<0.001), and a significant day by group interaction (F (1,22) = 10.54, p<0.01). Further analysis (one-way ANOVA) found that the cocaine group increased their activity from day 1 to day 6 (F (1,11) = 11.33, p<0.01), but found no such effect for the vehicle group (F (1, 11) = 0.183, p>0.05).

Interestingly, while general activity levels increased over days for the cocaine group, the rate of entry into the magazine did not (see Figure 5b). A mixed ANOVA with day (1–6) as the within subjects factor and group as the between subjects factor showed a significant main effect of day (F (5,110) = 4.77, p = 0.001), reflecting a decrease in entries over days for both groups, since there was no significant main effect of group (F (1,22) = 0.002, p>0.05) or day by group interaction (F (5,110) = 0.49, p>0.05). Magazine entry behavior was also recorded during the cocaine exposure phase of experiment 1, and analysis of these data revealed a strikingly different pattern of results. In contrast to the naïve rats in experiment 2, rats in experiment 1 already had considerable experience collecting food rewards from the magazine before the cocaine exposure phase, and so during these sessions the magazine entry response was likely to represent an instance of conditioned food-motivated behavior. In this set of animals, an increase in magazine entries was observed over days for the cocaine group, but not for the vehicle group, as displayed in Figure 5c. A mixed ANOVA detected a main effect of day (F (5,100) = 2.48, p<0.05), a main effect of group (F (1,20) = 4.60, p<0.05), and a day by group interaction (F (5,100) = 2.58, p<0.05). Further testing (one way ANOVA) found a main effect of day for the cocaine group (F (5, 50) = 2.35, p = 0.05), reflecting an increase in magazine entries over days. This increase appears to taper off after day 4, perhaps due to extinction of the conditioned approach response, since no food rewards were delivered during this phase of the experiment. In contrast, although a significant main effect of day was also detected for the vehicle group (F (5,50) = 7.46, p<0.001), this result reflected a drop in magazine entries over days, again, presumably due to extinction of this behavior.

## Discussion

In this study we sought to determine if repeated exposure to experimenter-delivered cocaine increases rats' tendency to seek out rewards when presented with a reward-paired cue and/or biases their tendency to acquire a habitual response selection strategy when pursuing a food reward. Our results support both hypotheses, demonstrating that repeated experimenter-delivered cocaine can 1) facilitate the expression of PIT for food reward, a relatively pure measure of cue-evoked incentive motivation, and 2) bias rats towards using a habitual control strategy when pursuing food reward. Furthermore, by using a within-subjects design to examine these behavioral phenomena, the current results indicate that the observed alterations in motivation and action selection can result from the same cocaine exposure regimen, and are therefore not likely to be particularly parameter dependent. While the additional time (5–7 d) between the PIT and outcome devaluation tests does not allow us to make a definitive statement about the dependence of these effects on the drug exposure-to-test interval, the current results do allow us to conclude that the cocaine exposure regimen used here results in an alteration of reward-seeking behavior that persists across a window of 15–22 d after the cessation of drug exposure.

Our findings are in line with previous reports that repeated peripheral administration of amphetamine facilitates expression of PIT [51], demonstrating that both psychostimulants have similar effects on cue-induced motivation despite differences in their mechanisms of action. This might be expected given demonstrations of behavioral cross-sensitization between these psychostimulants [52]–[54]. Thus, despite their differential effects and modes of action on the dopamine system [35]–[36], these drugs appear to share the ability to produce a nonspecific enhancement in cue-evoked incentive motivation. A more recent study [55] found that rats trained to self-administer intravenous cocaine exhibited an enhancement in PIT. Our results demonstrate that such an effect can also be produced with other modes of drug delivery, which supports the validity of using experimenter-delivered cocaine administration to model this consequence of cocaine taking. However, given recent findings that passive and self-regulated cocaine intake have different neurochemical effects [56], [57] and support distinct adaptations in the circuitry controlling dopamine signaling [58], [59], further comparison of the effects of these treatments seems warranted.

In this experiment we have used the PIT task to assay cue-elicited incentive motivation. In this task, Pavlovian cues and instrumental actions are trained during separate phases of the experiment, preventing these events from becoming directly associated with one another. Because of the design of this task, any increase in responding occurring during the reward-paired cue at test must be due to its incentive motivational properties, acquired during the Pavlovian conditioning phase. Furthermore, because the reward-paired cue is noncontingently presented at test, its behavioral effects represent the elicitation or invigoration of reward seeking behavior, as opposed to a conditioned reinforcement effect. Using a PIT design much like that used here, we have recently shown that rats trained to self-administer cocaine increase their pursuit of cocaine when presented with a cocaine-paired cue [60]. Although this finding demonstrates that Pavlovian incentive motivational processes contribute to instrumental drug motivated behavior, the role of cocaine *sensitization* in this phenomenon is difficult to isolate from that drug's more direct role as a behavioral goal or reinforcer. Because the subject is repeatedly exposed to the drug during instrumental and Pavlovian conditioning sessions, it is not clear whether or to what degree the cue's effects on drug seeking have been altered by drug-induced sensitization. As discussed earlier, the nonspecific effects of repeated drug exposure on motivation are easier to evaluate when the target response is reinforced by a different reward, like palatable food, because in this case the drug exposure treatment is not conflated with its function as a reinforcer. The current findings add to a growing literature showing that repeated drug administration can enhance appetitive behaviors generated by non-drug rewards. [29], [30], [34]–[37], [51], [61]–[65]. Such findings suggest that extended drug exposure produces nonspecific alterations in motivation, perhaps via adaptations in the dopamine system. Indeed, while it is firmly established that repeated drug exposure can sensitize the dopamine response to future drug challenges, there is also growing evidence that such treatment generates broad cross-sensitization of dopamine release to other drugs [66]–[68], as well as to chemical stimulation [69] and natural reward stimuli and associated cues [70], [71]. Linking these changes in dopamine signaling to alterations in incentive motivation will require further research.

We found further evidence of the motivational impact of cocaine treatment during the sensitization phase of experiment 1, in that repeated cocaine treatments elicited an increase in magazine approach behavior. Importantly, this effect was not simply due to an increase in locomotor behavior; no such effect was observed in rats that were unfamiliar with the behavioral chamber (for whom the magazine approach response was most likely to be a simple exploratory behavior), even though these rats did show evidence of sensitization of their general locomotor activity. Thus, the tendency for repeated cocaine exposure to enhance magazine approach behavior depends on whether or not that activity is motivated by food reward. Although this behavioral measure does not provide the kind of pure assessment of cue-evoked incentive motivation that PIT provides, this finding does bolster the view that repeated cocaine exposure sensitizes motivated behavior.

Our results also provide evidence that repeated cocaine exposure promotes use of a habitual, rather than a goal-directed, response strategy, which follows with the suggestion of others that drug-induced sensitization facilitates acquisition of habitual control [29]. This enhancement in S-R learning should leave drug-seeking behavior less sensitive to its various negative consequences or to the desire to abstain. It is possible that repeated exposure to cocaine and other drugs could impact the action selection strategies more generally. Our results suggest that cocaine can support such an effect, demonstrating that repeated cocaine administration enhances habitual control during the pursuit of natural rewards. Similar findings have been obtained by pretreating rats with amphetamine [29], [30], indicating that these psychostimulants also have in common the ability to bias action selection towards habitual control.

It has been argued that habitual control, which is typically tested in extinction (without negative feedback), does not adequately model the compulsive nature of drug-seeking behavior, which tends to persist despite its negative consequences [13], [27], [72]. However, in the current study, cocaine-treated rats also appeared to have some difficulty suppressing their instrumental performance even when given response-contingent negative feedback (i.e., the opportunity to consume the devalued reward). It should be noted, however, that we did not detect a significant group difference in the sensitivity of lever pressing to outcome devaluation during this rewarded test phase, and so it is not possible to draw strong conclusions about whether or not the cocaine group's performance should be considered abnormal. Other findings suggest extensive or chronic access to cocaine is required for the development of compulsive forms of cocaine seeking in rodents [73]–[75], including behavior that is relatively insensitive to negative feedback (response extinction, electric footshock or shock-paired cues) [20], [21]. We might therefore expect an even clearer disruption of outcome devaluation sensitivity during these tests (particularly the rewarded test) in rats given more frequent cocaine exposures or larger doses. However, since these other studies used response-contingent intravenous cocaine exposure, the mode of cocaine delivery may also be an important factor determining the long-term impact of cocaine on behavioral control. Future studies should investigate these possibilities.

It should be noted that, in the current study, rats were placed in the chamber used for behavioral training and testing after each daily cocaine (or saline) treatment. Although we did not explore this issue here, it seems likely that drug-induced context conditioning contributed to the behavioral effects that we observed in cocaine-treated rats. For instance, context-mediated learning has been shown to play an important role in other forms of behavioral sensitization [76]–[78]. Furthermore, a recent study found that cocaine-paired contextual stimuli can provoke impulsive decision making in rats [79]. We have also recently shown that contextual cues paired with alcohol intoxication produce a transient disruption in instrumental control, causing rats to shift from goal-directed to habitual performance [31]. Thus, the impact of repeated cocaine exposure on PIT and outcome devaluation performance may be mediated, at least in part, by context-cocaine conditioning. Future studies should explore this possibility more directly.

Determining how aberrations in habitual control and incentive motivation work together to generate compulsive drug seeking is an important goal for future research. One interesting possibility is that these processes make distinct, stage-dependent contributions to the development of addiction. They may also affect different components of drug-related behavior. For instance, it has been argued that while exaggerated habits may contribute to drug-taking or consumption, it is the sensitization of incentive motivation that maintains compulsive drug-seeking and disrupts attempts to abstain [2]. Conversely, studies have shown that compulsive drug-seeking behavior can be induced independently of any increased motivation for the drug, by producing habitual control of behavior [21], [80], [81], leading some to speculate that it is in fact increased incentive motivation for the drug that develops first, but it is the habitual, S-R action selection strategy that facilitates compulsive drug-seeking during the later stages of addiction [82]. Interestingly, basic behavioral research has shown that reward-paired cues tend to facilitate performance by engaging habits [83], [84], suggesting that these two processes work in tandem to control behavior. Our data show that, within a single set of rats, cocaine administration can sensitize both cue-evoked incentive motivation and habit formation, which is clearly compatible with this view. Finally, while this work suggests that drug-induced aberrations in motivation and behavioral control may contribute to addiction, the complex characteristics of this condition would seem to suggest that other cognitive and behavioral dysfunctions, such as alterations in prefrontal cortical areas and executive control, also play an important role.
